# RETRACTED: Rahman et al. An Overview of Antimicrobial Stewardship Optimization: The Use of Antibiotics in Humans and Animals to Prevent Resistance. *Antibiotics* 2022, *11*, 667

**DOI:** 10.3390/antibiotics15020200

**Published:** 2026-02-12

**Authors:** Md. Mominur Rahman, Mst. Afroza Alam Tumpa, Mehrukh Zehravi, Md. Taslim Sarker, Md. Yamin, Md. Rezaul Islam, Md. Harun-Or-Rashid, Muniruddin Ahmed, Sarker Ramproshad, Banani Mondal, Abhijit Dey, Fouad Damiri, Mohammed Berrada, Md. Habibur Rahman, Simona Cavalu

**Affiliations:** 1 Department of Pharmacy, Faculty of Allied Health Sciences, Daffodil International University, Dhaka 1207, Bangladesh; afrozaalm2@gmail.com (M.A.A.T.); taslim29-136@diu.edu.bd (M.T.S.); yamin29-1313@diu.edu.bd (M.Y.); rezaul29-1301@diu.edu.bd (M.R.I.); harun.1947.1952@gmail.com (M.H.-O.-R.); drmuniruddin@gmail.com (M.A.); 2 Department of Clinical Pharmacy Girls Section, Prince Sattam Bin Abdul Aziz University, Alkharj 11942, Saudi Arabia; mahrukh.zehravi@hotmail.com; 3 Department of Pharmacy, Ranada Prasad Shaha University, Narayanganj 1400, Bangladesh; ramproshad131135@gmail.com (S.R.); banani091110@gmail.com (B.M.); 4 Department of Life Sciences, Presidency University, Kolkata 700073, India; abhijit.dbs@presiuniv.ac.in; 5 Labortory of Biomolecules and Organic Synthesis (BioSynthO), Department of Chemistry, Faculty of Sciences Ben M’Sick, University Hassan II of Casablanca, Casablanca 20000, Morocco; fouad.damiri@outlook.fr (F.D.); berrada_moh@hotmail.com (M.B.); 6 Department of Global Medical Science, Wonju College of Medicine, Yonsei University, Wonju 26426, Republic of Korea; 7 Faculty of Medicine and Pharmacy, University of Oradea, P-ta 1 Decembrie 10, 410087 Oradea, Romania

The journal retracts the article “An Overview of Antimicrobial Stewardship Optimization: The Use of Antibiotics in Humans and Animals to Prevent Resistance” [1], cited above.

Following publication, concerns were brought to the attention of the Editorial Office regarding an overlap between this publication [1], and earlier article, and reviews [2,3,4] produced by different groups of authors.

Adhering to our standard procedure, an investigation was conducted by the Editorial Office and Editorial Board, confirming a substantial overlap of text, reference order, and a figure between this study and the abovementioned publications [2,3,4]. Following discussions, the authors collaborated but were unable to satisfactorily address the initially raised concerns. Consequently, the Editorial Board has lost confidence in the originality of the findings and author contributions and has decided to retract this publication [1], as per MDPI’s retraction policy (https://www.mdpi.com/ethics#_bookmark30).

This retraction was approved by the Editor-in-Chief of the journal *Antibiotics*.

Simona Cavalu and Fouad Damiri did not agree to this retraction. The remaining authors did not provide a comment on this decision.
